# Venous excess ultrasound score association with acute kidney injury in critically ill patients: a systematic review and meta-analysis of observational studies

**DOI:** 10.1186/s13089-025-00413-9

**Published:** 2025-03-03

**Authors:** Rafael Hortêncio Melo, Luciana Gioli-Pereira, Edielle Melo, Philippe Rola

**Affiliations:** 1 Hospital Municipal Vila Santa Catarina Dr.Gilson de Cássia Marques de Carvalho; Hospital Israelita Albert Einstein, Av. Santa Catarina, 2785 - Vila Santa Catarina, São Paulo, SP Brazil; 2 University of Montreal, Montreal, Canada

**Keywords:** Fluid resuscitation, Venous congestion, Fluid responsiveness, Critical care, VExUS, Fluid tolerance

## Abstract

**Background:**

Systemic venous congestion assessed by the venous excess ultrasound score (VExUS), has been associated with acute kidney injury (AKI) in patients undergoing cardiac surgery. However, there is a lack of evidence of this association in the general critically ill patients.

**Study Design and Methods:**

PubMed, Embase, and Cochrane databases were searched for observational prospective studies that included critically ill patients and analyzed VExUS score on the first day of admission to the ICU. The main outcome was occurrence of AKI. Secondary outcome was all-cause mortality. Statistical analysis was performed using Review Manager 5.4.1. Odds ratios (OR) with 95% confidence interval were pooled using a random-effects model. The Quality in Prognosis Studies (QUIPS) tool was used to assess risk of bias. Publication bias was assessed via funnel plot and heterogeneity was examined with I^2^ statistics.

**Results:**

Our analysis included 1036 patients from nine studies, of whom 17.4% presented venous congestion according to VExUS definition. In critically ill patients presenting with venous congestion (VExUS score ≥ 2), the incidence of AKI was significantly higher as compared with those without congestion (OR 2.63, 95% CI 1.06–6.54; p = 0.04; I^2^ = 74%). The association was notably stronger in cardiac surgery patients (OR 3.86, 95% CI 2.32–6.42; p < 0.00001; i^2^ = 0%). There was no significant association between venous congestion and all-cause mortality (OR 1.25, 95% CI 0.71–2.19; p = 0.44; i^2^ = 8%).

**Conclusions:**

These findings suggest that VExUS score may correlate with an elevation in the incidence AKI in critically ill patients, with a more pronounced effect observed within the subgroup of patients undergoing cardiac surgery. There was no statistically significant association between VExUS score and all-cause mortality.

*Clinical Trial Registration*: PROSPERO under protocol number CRD535513.

**Supplementary Information:**

The online version contains supplementary material available at 10.1186/s13089-025-00413-9.

## Introduction

While intravenous fluid infusion has long been a cornerstone of resuscitation, there is growing evidence of organ dysfunction due to fluid overload in critically ill patients [1–4]. Venous congestion, whether at the splanchnic or pulmonary level, is probably the main component responsible for the harmful effects of hypervolemia, presumably by the reduction of perfusion pressure, with the encapsulated organs, like the kidneys, the most sensitive to its effects [5].

In the past, central venous pressure (CVP) was interpreted as a surrogate for venous congestion, and its association with acute kidney injury (AKI) is well known [6]. However, CVP can only be measured invasively, and may not offer the same level of organ-based perspective that is potentially required. There are also technical measurement issues that can result in a certain margin of error [7, 8]. Therefore, it has a limited role in the identification of venous congestion in many patients [9].

The venous excess ultrasound (VExUS) score is a novel and promising method that grades the Doppler waves of splanchnic encapsulated organs (e.g.,liver and kidney) starting from a dilated inferior vena cava (IVC) using point-of-care ultrasound (POCUS) that has shown a greater association with AKI than CVP in cardiac surgery patients [10]. Even so, whether VExUS should be preferred for assessing venous congestion remains unknown, given the lack of randomized data. Therefore, we performed a systematic review and meta-analysis to evaluate the association of the VExUS score with AKI in critically ill patients.

## Study design and methods

This systematic review and meta-analysis was performed and reported in accordance with the Cochrane Collaboration Handbook for Systematic Review of Interventions and the Preferred Reporting Items for Systematic Reviews and Meta-Analysis (PRISMA) Statement guidelines [11, 12]. As such, our protocol was registered on PROSPERO on April 19, 2024, under protocol number CRD535513.

### Eligibility criteria

Inclusion in this meta-analysis was restricted to studies that met all the following eligibility criteria: (1) prospective and cross-sectional studies; (2) assessing VExUS at least on the first day of admission in ICU; (3) enrolling critically ill patients; and (4) reported at least one outcome of interest. We excluded (1) preclinical studies; (2) studies including pediatric patients (3) case reports and conference abstracts; (4) non-english articles.

We defined critically ill patients as any patient with the need of admission in an ICU for either organ monitoring or support.

### Search strategy and data extraction

We systematically searched PubMed, Embase, and Cochrane Library databases from inception through the final search date of April 30, 2024. We used the following search terms: ‘critically ill’, ‘ICU’, ‘critical care’,’vexus’,’venous congestion’,’vexus score’, ‘acute kidney injury’ and ‘kidney failure’. The complete search strategy is available in the Supplementary Appendix. Two authors (R.H.M., L.G.P.) independently extracted the available study characteristics, event rates, and/or adjusted odds ratios (OR) from full-text published articles following prespecified search criteria and quality assessment. Zotero software (version 6.0.36) helped to exclude duplicate studies. Additionally, a backward search (snowballing) and a forward search (citation-tracking) were conducted for the included articles and relevant literature review. If the required data were not available in the published studies, we contacted the corresponding author to obtain the information. Authors were contacted for any additional data not sufficiently reported in the publication. Disagreements were solved by discussion with a third author (E.M.).

### Endpoints

The main outcome was the occurrence of AKI by Kidney Disease Improving Global Outcomes (KDIGO) definition. The secondary outcome was all-cause mortality. Venous congestion was defined as VExUS ≥ 2 according to the original investigation by Beuabien-Souligny and colleagues [10]. Prespecified subgroup analyses included data restricted to cardiac surgery patients and general critically ill patients.

### Quality assessment

Two investigators (R.H.M., L.G.P.) independently assessed the quality of included studies using the Quality in Prognosis Studies (QUIPS) tool for prognostic studies, which allows labeling studies as of low, moderate, or high risk of bias in six domains: study participation, study attrition, prognostic factor measurement, outcome measurement, study confounding, and statistical analysis and reporting [13]. Discrepancies were solved through consensus. In addition, we assessed small studies effect (publication bias) through funnel plot analysis for the main outcome.

### Statistical analysis

Pooled treatment effects for binary endpoints were compared using odds ratio (OR) with 95% confidence intervals (CI) and p-values less than 0.05 were deemed significant for treatment effects. Cochran Q test and I^2^ statistics were used to assess for heterogeneity; p-values inferior to 0.10 and I^2^ > 25% were considered significant for heterogeneity. We used a DerSimonian and Laird random-effects model accounting for heterogeneity among studies. Statistical analysis was performed using Review Manager 5.4.1 (Cochrane Center, The Cochrane Collaboration, Denmark).

## Results

### Study selection and characteristics

**Fig. 1:**
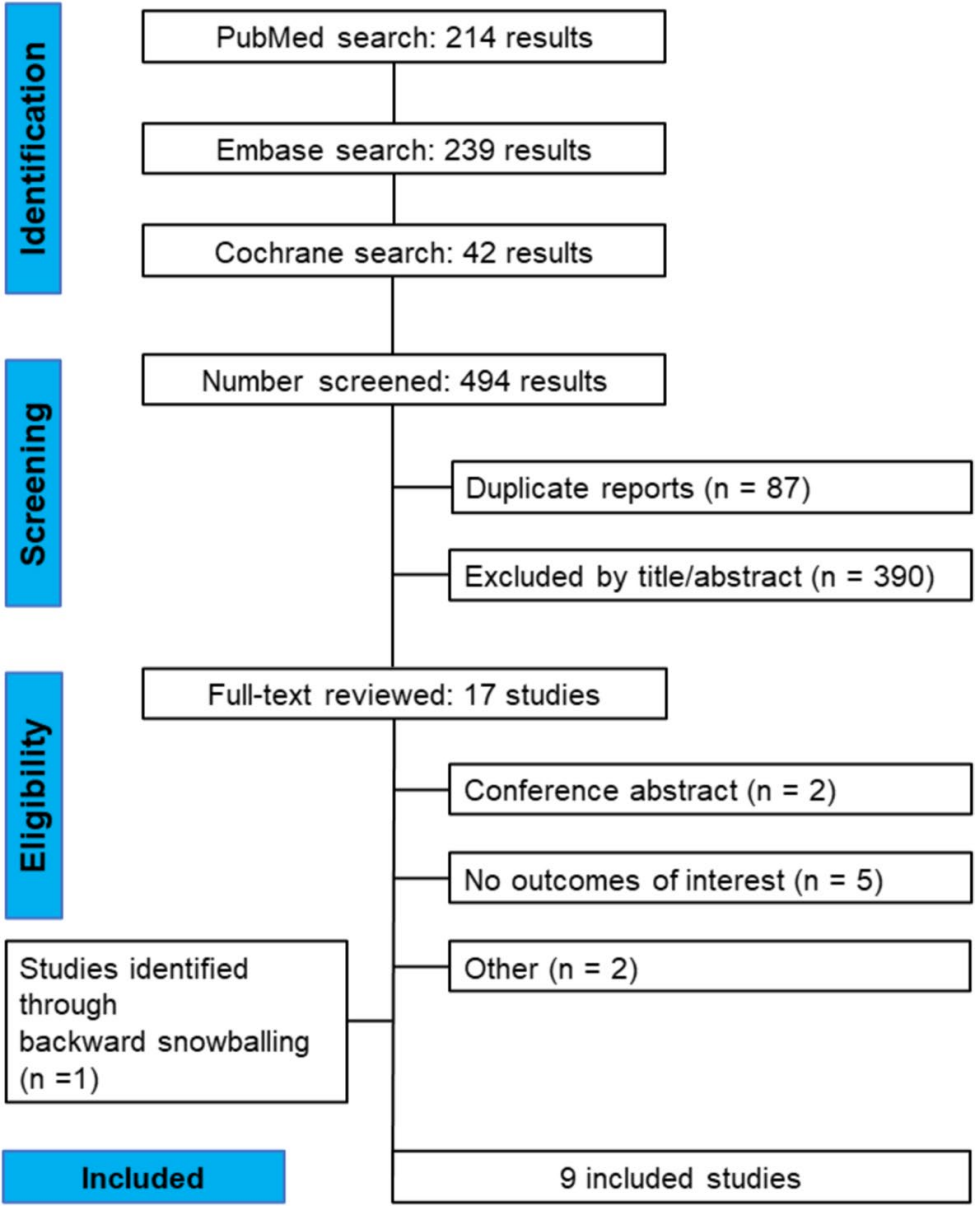
PRISMA flow diagram of study screening and selection

As detailed in Fig. 1, the initial search yielded 494 results. After the removal of duplicate records and ineligible studies, 17 remained and were fully reviewed based on inclusion criteria. After further examination, nine publications were excluded. One study was included after backward research, resulting in nine studies, comprising 1036 patients. Three studies included exclusively cardiac surgery patients; one study included only patients with ongoing acute coronary syndromes; and the remaining trials included general critically ill patients.

The mean age was 62.7 years, 385 (37.1%) were women, and the prevalence of venous congestion on ICU admission ranged from 1.7% to 27%. Preexisting chronic kidney disease (CKD) varied from 3% to 25.5% and was not reported in two studies [14, 15]. Detailed baseline characteristics of included trials are displayed in Table 1.

**Table 1:** – Individual study characteristics

Study	Type of Analysis	Population	Nº of Patients	Age (Years), median	Female, n (%)	CKD, n (%)	Low LVEF or CHF, n (%)	VExUS 0–1, n (%)	VExUS ≥ 2, n (%)	Incidence of AKI, n (%)
Andrei, 2023	Prospective cohort, multicenter	ICU	145	64	59 (41)	15 (10)	21 (15)	116 (80)	29 (20)	68 (47)
Beaubien-Souligny, 2020	Prospective cohort, single center	Cardiac surgery	145	66	38 (26.2)	37 (25.5)	31 (21.4)	113 (78)	32 (22)	49 (33.8)
Beaubien-Souligny, 2024	Prospective cohort, multicenter	ICU	125	67	35 (27)	22 (17.6)	21 (16.8)	83 (66.4)	30 (24)	NA
Li, 2024	Prospective cohort, single center	Cardiac surgery	230	59.7	95 (41.3)	NA	NA	197 (85.6)	33 (14.3)	53 (23)
Munoz, 2024	Cross-sectional, multicenter	ICU	90	63	49 (51)	NA	NA	82 (91.1)	8 (8.9)	44 (48.8)
Prager, 2024	Prospective cohort, multicenter	ICU septic shock	75	64.7	39 (52)	8 (10.6)	14 (18.6)	61 (81)	14 (19)	NA
Trigkidis, 2024	Prospective cohort, single center	ICU	89	62	34 (38)	3 (3)	NA	65 (73)	24 (27)	20 (22)
Utrilla-Alvarez, 2023	Cross-sectional, single center	Cardiac surgery	60	60	25 (41.7)	4 (6.7)	16 (26.6)	59 (98.3)	1 (1.7%)	21 (35)
Viana-Rojas, 2023	Prospective cohort, single center	ACS	77	58.3	11 (14.3)	5 (6.4)	23 (29.8)	67 (87%)	10 (13)	19 (24.6)

**Fig. 2:**
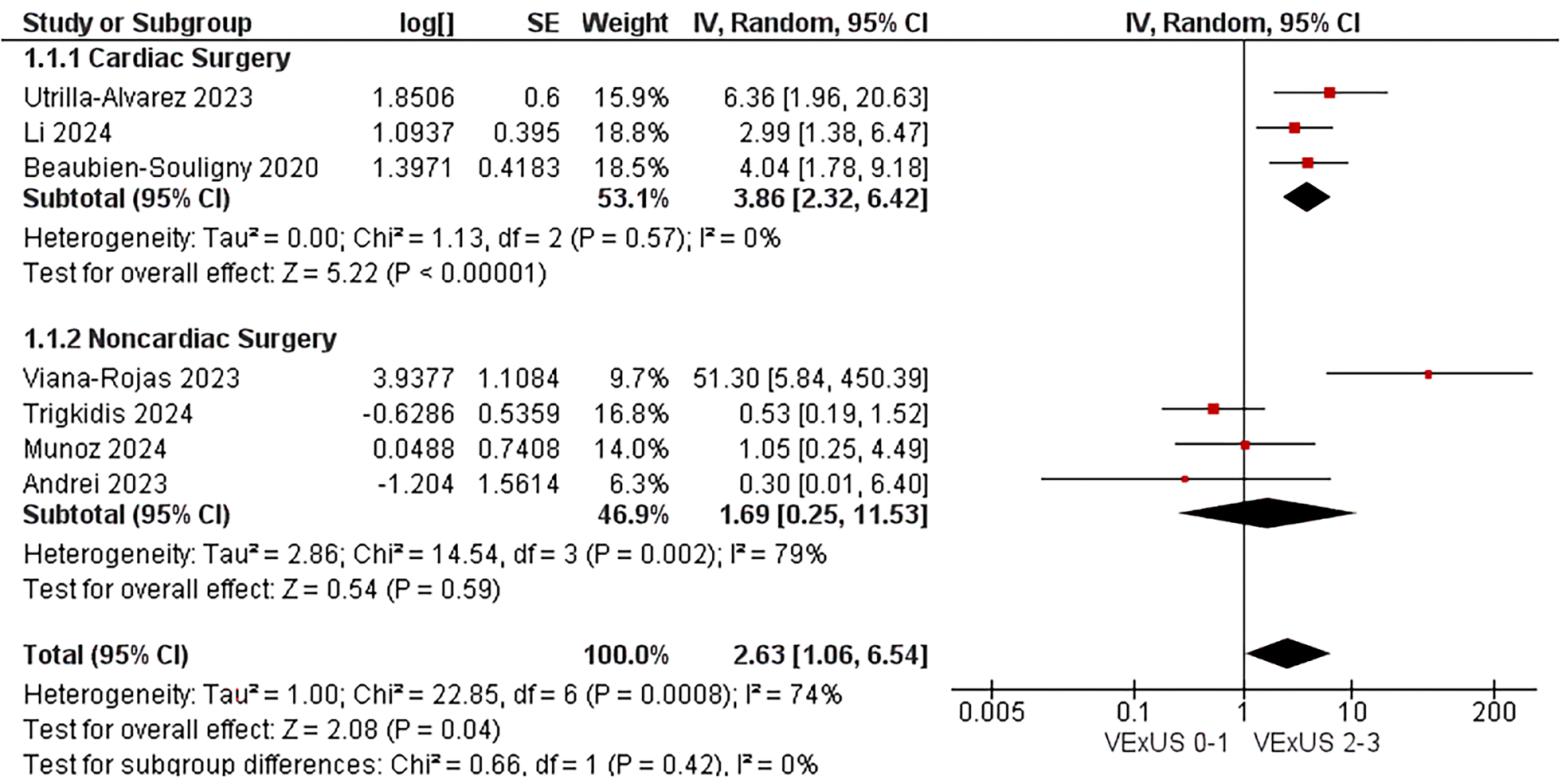
VExUS score association with AKI in the critically ill patients

**Fig. 3:**
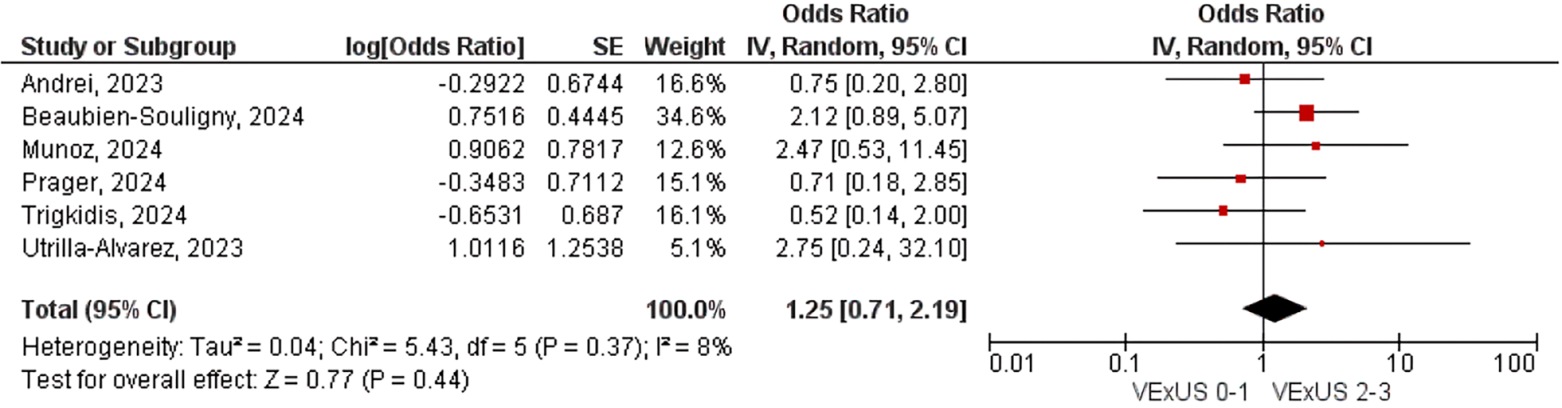
VExUS was not associated with all-cause mortality

The combined study population demonstrated a statically significant association of VExUS score ≥ 2 on admission with occurrence of AKI (OR 2.63; 95% CI 1·06–6·54; p = 0.02; I^2^ = 74%; Fig. 2). In a sub-analysis of non-cardiac surgery subgroup, venous congestion was not associated with AKI (OR 1.69; 95% CI 0·25–11·53; p = 0.59; I^2^ = 79%). In contrast, the subgroup of cardiac surgery patients showed a higher correlation with AKI (OR 3.86; 95% CI 2·32–6·42; p < 0.00001; I^2^ = 0%; Fig. 2). There was no significant interaction between subgroups (p = 0.21). All-cause mortality was not available in three studies, and was not associated with VExUS (OR 1.25; 95% CI 0·71–2·19; p = 0.44; I^2^ = 8% Fig. 3).

### Quality assessment

**Fig. 4:**
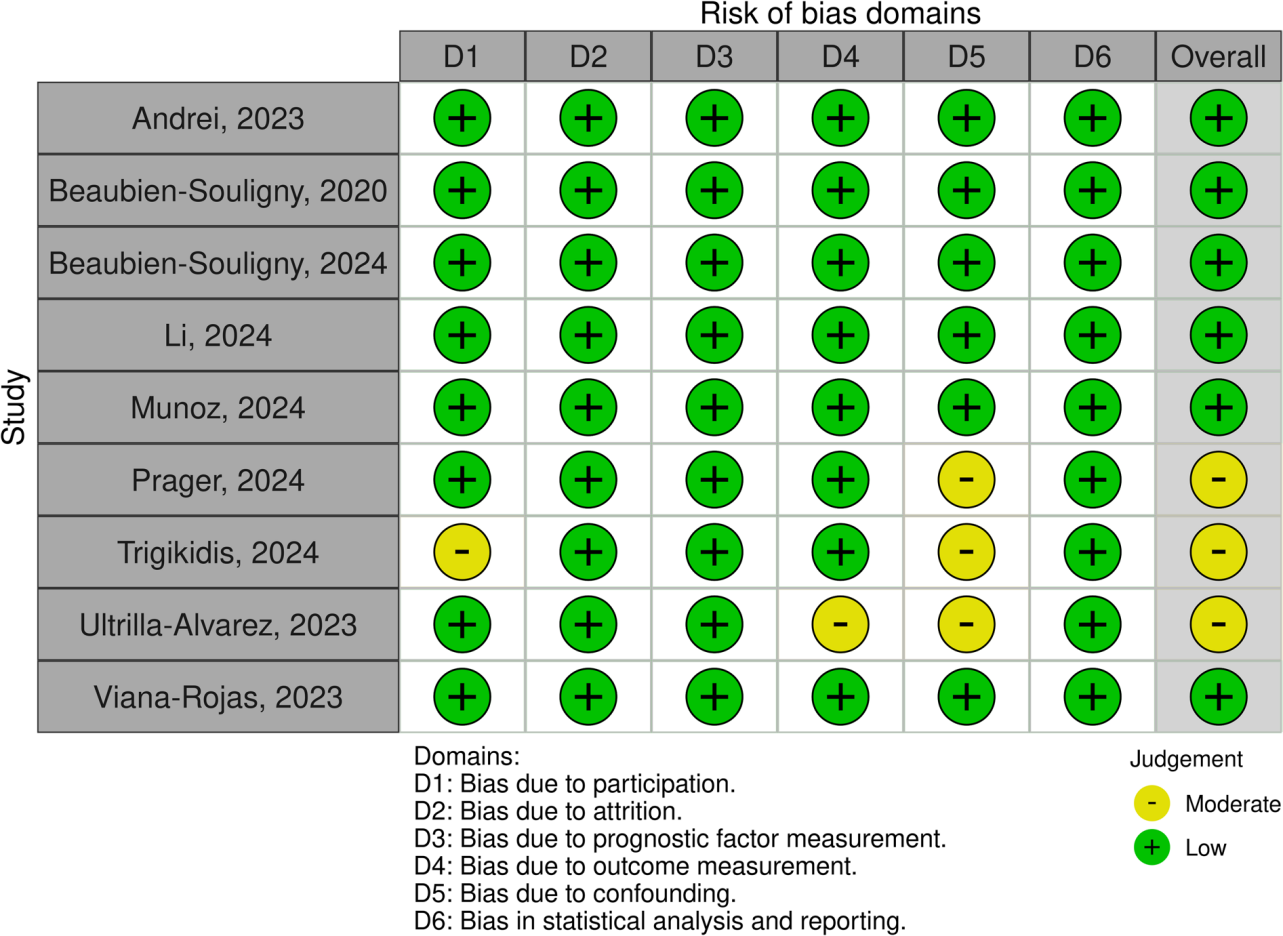
Individual study appraisal of all QUIPS domains

Three studies presented with overall moderate risk of bias [16–18]. The remaining articles were considered with overall low risk of bias. Individual study appraisal of all domains is shown in Fig. 4. Funnel plot showed slightly asymmetry (Supplementary Fig. 5) implying small study effect, but Egger regression test could not be performed due to the limited number of studies.

## Discussion

In this meta-analysis encompassing 1036 patients from nine studies, we compared the incidence of AKI in critically ill patients with versus without venous congestion defined by VExUS score. Venous congestion was associated with increased occurrence of AKI, but not with all-cause mortality. There was no significant interaction between subgroups of cardiac surgery and non-cardiac surgery.

Systemic venous congestion is one of the components of the emerging concept of fluid tolerance (FT) [19, 20], and its evaluation is becoming a matter of interest during resuscitation [21].FT is defined as the capacity of different organs to tolerate fluid administration without causing or worsening organ dysfunction, and it is determined by many factors such as age, structural heart disease, illness severity and glycocalyx dysfunction [22, 23]. FT should be evaluated in two different hemodynamic compartments: (1) left, considering the left heart filling pressures and the degree of pulmonary congestion; and (2) right, assessing the right heart filling pressures and the degree of fluid overload in the systemic venous compartment.

There are multiple mechanisms by which venous congestion could cause organ dysfunction. In the setting of venous congestion, the increase in venous hydrostatic pressure could reduce organ perfusion pressure. Venous congestion can also lead to hemodilution due to hypervolemia and an increase in the diffusion distance of red blood cells, impairing the microcirculation [24]. Over the years, CVP has been used as an indication of venous congestion. However, this method has technical challenges and requires invasive catheters. Additionally, CVP is affected by the interaction between venous return and ventricular function. It is also influenced by increased intrathoracic pressure in conditions such as pneumothorax, cardiac tamponade, and the use of positive end-expiratory pressure (PEEP). Therefore, it has a limited role in assessing right FT.

The critical care physician has a varied range of tools available to assess both compartments of FT. In the ICU, rapid hemodynamic assessment is required. Thus, POCUS is becoming an increasingly valuable tool in this scenario, with the potential for wide application in assessing both right and left FT. Even though venous congestion estimated by Doppler in the splanchnic circulation has already been studied over the past years and has shown association with clinical outcomes [25–28], it was only after the creation of an ultrasound score by Beaubien-Souligny and colleagues [10] that it became standardized. This technique is a novel combination of Doppler wave patterns of splanchnic circulation that graded venous congestion in a numerical score named VExUS. This scoring system showed a positive likelihood ratio of 6.37 for the development of cardiorenal AKI [10]. Although this finding generated a lot of interest among intensive care physicians and its physiologic plausibility [29, 30], it was validated only in cardiac surgery patients. Few trials have been performed in other subgroups of critically ill patients [31–33], and there are many discrepancies in the population, size and methodology among these studies. Therefore, there is a lack of strong evidence correlating VExUS with AKI in the general ICU patients.

To the best of our knowledge, this is the first meta-analysis to focus on the occurrence of AKI in critically ill patients with versus without venous congestion defined by VExUS score. Overall, our results suggest that VExUS ≥ 2 on admission is associated with occurrence of AKI especially in cardiac surgery patients.

Our study has limitations. First, our analysis included only observational studies, combining both cross-sectional and prospective designs. This pooling introduces potential confounding and inherent bias due to the distinct nature of each study type. Second, we only evaluated the correlation of VExUS at ICU admission, and not after 72 hours, which could mitigate the impact of venous congestion on clinical outcomes [17]. Third, significant heterogeneity (I^2^ = 74%) was observed among the included studies, suggesting that the results should be interpreted with caution. This heterogeneity may stem from variations in patient populations, as the studies included a wide spectrum of critically ill patients, ranging from those undergoing cardiac surgery to those with acute coronary syndromes and sepsis. Such clinical diversity likely influences the incidence and mechanisms of AKI as well as responses to fluid management and venous congestion. Fourth, a subgroup analysis of non-cardiac surgery patients indicated no correlation between the VExUS score and the incidence of AKI. However the interaction between subgroups was not statistically significant (p = 0.42), suggesting that the absence of a significant association between the VExUS score and AKI in non-cardiac surgery patients may be partially attributed to insufficient statistical power. Nevertheless alternative mechanisms of AKI in the general ICU patients—such as sepsis, nephrotoxicity, and organ crosstalk [34–36]—might also account for these findings. In this group, venous congestion is likely a minor contributor to the development of AKI compared to cardiac surgery patients. In the latter population, although the etiology is multifactorial, cardiorenal syndrome, which is typically associated with venous congestion, tends to be more prevalent [37]. Finally, we were unable to statistically analyze other relevant outcomes, such as ventilation-free days and ICU length of stay, due to incomplete reporting and the absence of individual-level patient data.

## Conclusion

In this meta-analysis of nine observational studies, venous congestion measured by the VExUS score was associated with an increased incidence of AKI in critically ill patients, particularly in the subgroup of cardiac surgery patients. There was no association between venous congestion and mortality. These findings should be interpreted considering its heterogeneous population, and additional studies are warranted to assess whether a VExUS-based management strategy can have a clinical impact and improve outcomes.

## Supplementary Information

Supplementary material. 1.

## Data Availability

The data that support the findings of this study are available from the main articles analyzed and described in manuscript and its supplementary materials.

## Abbreviations

AKI: Acute kidney injury
CI: Confidence interval
CVP: Central venous pressure
FT: Fluid tolerance
ICU: Intensive care unit
IVC: Inferior vena cava
KDIGO: Kidney disease improving global outcomes
OR: Odds ratio
PEEP: Positive end expiratory pressure
POCUS: Point-of-care ultrasound
PRISMA: Preferred reporting items for systematic reviews and meta-analyses
PROSPERO: Prospective register of systematic reviews
QUIPS: Quality in prognosis studies
VExUS: Venous excess ultrasound
